# Physiological responses and external validity of a new setting for taekwondo combat simulation

**DOI:** 10.1371/journal.pone.0171553

**Published:** 2017-02-03

**Authors:** Matheus Hausen, Pedro Paulo Soares, Marcus Paulo Araújo, Flávia Porto, Emerson Franchini, Craig Alan Bridge, Jonas Gurgel

**Affiliations:** 1 Graduate Program on Cardiovascular Sciences, Medical Science Center, Fluminense Federal University, Niterói, Brazil; 2 Biomechanics Research Group, Fluminense Federal University, Niterói, Brazil; 3 Physical Education and Sports Institute, Rio de Janeiro State University, Rio de Janeiro, Brazil; 4 Martial Arts and Combat Sports Research Group, School of Physical Education and Sport, University of São Paulo, São Paulo, Brazil; 5 Sport and Exercise Research Group, Department of Sport and Physical Activity, Edge Hill University, Ormskirk, United Kingdom; University of Utah, UNITED STATES

## Abstract

Combat simulations have served as an alternative framework to study the cardiorespiratory demands of the activity in combat sports, but this setting imposes rule-restrictions that may compromise the competitiveness of the bouts. The aim of this study was to assess the cardiorespiratory responses to a full-contact taekwondo combat simulation using a safe and externally valid competitive setting. Twelve male national level taekwondo athletes visited the laboratory on two separate occasions. On the first visit, anthropometric and running cardiopulmonary exercise assessments were performed. In the following two to seven days, participants performed a full-contact combat simulation, using a specifically designed gas analyser protector. Oxygen uptake ($\dot{\text{V}}{\text{O}}_{2}$), heart rate (HR) and capillary blood lactate measurements ([La^-^]) were obtained. Time-motion analysis was performed to compare activity profile. The simulation yielded broadly comparable activity profiles to those performed in competition, a mean $\dot{\text{V}}{\text{O}}_{2}$ of 36.6 ± 3.9 ml.kg^-1^.min^-1^ (73 ± 6% $\dot{\text{V}}{\text{O}}_{2\text{PEAK}}$) and mean HR of 177 ± 10 beats.min^-1^ (93 ± 5% HR_PEAK_). A peak $\dot{\text{V}}{\text{O}}_{2}$ of 44.8 ± 5.0 ml.kg^-1^.min^-1^ (89 ± 5% $\dot{\text{V}}{\text{O}}_{2\text{PEAK}}$), a peak heart rate of 190 ± 13 beats.min^-1^ (98 ± 3% HRmax) and peak [La^-^] of 12.3 ± 2.9 mmol.L^–1^ was elicited by the bouts. Regarding time-motion analysis, combat simulation presented a similar exchange time, a shorter preparation time and a longer exchange-preparation ratio. Taekwondo combats capturing the full-contact competitive elements of a bout elicit moderate to high cardiorespiratory demands on the competitors. These data are valuable to assist preparatory strategies within the sport.

## Introduction

Incorporating a full-contact striking style, taekwondo is characterized as an intermittent sport [1–3], demanding contributions from both aerobic and anaerobic pathways [4]. Thus, taekwondo athletes exhibit moderate to high cardiorespiratory fitness [5], which is desirable to support demands of competition matches [3,6], training sessions [7,8] and seems to provide a suitable recovery between high-intensity intermittent efforts [9]. However, the characterisation of the dynamics of these metabolic pathways during actual taekwondo combat remains unclear due to constraints associated with collecting physiological measurements in real competition [3]. To circumvent these constraints and provide broader insights into the energetic requirements of the activity, some research groups have collected physiological indices during simulated combat [7,4]. Two experimental paradigms have been favoured depending on the specific research objectives, and may be categorised as either ‘combat simulations’ or ‘combat-specific exercise protocols’ according to their characteristics [3]. Combat simulations involve the interaction of two opponents during a simulated fight, theoretically optimising the external validity of the activity and the energetic requirements. Whereas combat specific exercise protocols, attempt to broadly replicate combat activity sequences in the absence of a competitor with the intent of providing greater experimental control over the environment to permit the study of interventions [10].

Since the assessment of the acute physiological responses in real competition is limited to indirect measurements, due to the risk of impairing the performance in crucial events and the constraints associated with the event regulations, few studies have analysed the cardiorespiratory responses to taekwondo competition [2,3,6] and combat simulations [7,4,11]. Unfortunately, these investigations did not measure oxygen uptake ($\dot{\text{V}}{\text{O}}_{2}$) nor did they replicate the actual combat pattern, since several rules changes have been made including new scoring values for spinning and high kicks, the minimum of 10s to attack and the use of an electronic scoring system [12].

Recently, a number of studies have examined the cardiorespiratory demands of combat simulations, reporting a mean $\dot{\text{V}}{\text{O}}_{2}$ between 34.5 ± 5.5 and 53.5 ± 5.9 mL.kg^-1^.min^-1^, a mean heart rate varying between 156 ± 9 and 177 ± 10 beats.min^-1^ and a peak blood lactate concentration varying from 7.0 ± 1.5 to approximately 10 mmol.L^-1^ [4,13]. These studies have omitted the competitive elements of the match and hence might underestimate the true metabolic cost of the event. Moreover, to preserve the gas analyser situated on the back of the participant, the absence of an official electronic scoring system and the cautious approach adopted by athletes could affect the competitiveness of the bouts and, consequently, the cardiovascular demand. Thus, a simulation may not elicit the higher psychophysiological stress to the competitiveness of an official match [3]. Competition has yielded significantly higher values of mean HR and [La^-^] than in stationary-target combat protocols (188 ± 8 beats.min^-1^ and 12.2 ± 4.6 mmol.L^-1^, against 172 ± 4 beats.min^-1^ and 3.6 ± 2.7 mmol.L^-1^, respectively) [3].

In addition to understanding the physiological demands, performance analysis must be considered to establish an updated ergonomic framework with a technical and tactical profile. Time-motion analysis was previously used to assess the external validity of a taekwondo-exercise protocol [3], to describe a combat simulation [4], and has been widely used to assess taekwondo performance [1,2]. However, the performance analysis evidence available is dated before the recent taekwondo rule adjustments and combat simulations without attack-defence interactions may not adequately reproduce the competition responses. Therefore, the aim of the present study was to assess the cardiorespiratory responses to a full-contact taekwondo combat simulation, with the aid of a gas analyser protector, which allowed a safe and externally valid athlete interaction during an actual taekwondo combat. It was hypothesized that the full-contact combat simulation may offer a more externally valid representation of the metabolic cost of fighting in official competition.

## Methods

### Participants

Twelve male World Taekwondo Federation (WTF) national level taekwondo athletes (age 20±2 years, body mass 67.5 ± 6.7 kg, height 175 ± 8.1 cm, $\dot{\text{V}}{\text{O}}_{2\text{PEAK}}$ 49.6 ± 2.8 mL.kg^-1^.min^-1^, black belt holders) visited the laboratory on two separate occasions. Participants were actively engaged in training programs, between 10h and 16h per week, and Brazilian Taekwondo Confederation official events, with a competitive experience ranging between 1 to 8 years. Before the experiment, all participants were informed about the test procedures and potential risks and signed an informed consent form. The study was approved by the University Research Ethics Committee (Opinion #765.698), according to the Brazilian National Health Council resolution #466/12. Athletes also completed a physical active readiness questionnaire (PAR-Q) [14] and presented no positive answers. The individual in this manuscript has given written informed consent (as outlined in PLOS consent form) to publish these case details.

### Experimental design

During the first visit, participants underwent a treadmill cardiopulmonary exercise test (CPET). On the second visit, they participated in a combat simulation. During CPET, peak $\dot{\text{V}}{\text{O}}_{2}$ and HR were assessed to enable the expression of the cardio-respiratory responses as a percentage of participants’ maximal aerobic power (e.g. $\%\dot{\text{V}}{\text{O}}_{2\text{PEAK}}$ and %HR_PEAK_). Visits were separated by two and seven days and occurred at the same time of the day [15]. All participants were familiarized with the equipment and assessment procedures prior to the experiment. Participants were requested to eat and drink water regularly as in their typical training weeks. Before CPET and simulation, participants underwent thirty-five minutes rest in supine position to elicit proper resting cardiorespiratory responses [16]. Both exercises were performed in a controlled laboratory environment (between 18 and 23°C).

### Cardiopulmonary exercise testing

CPET was set up to last ten minutes using an individualized ramp protocol, with an initial speed of 60% of the maximum speed at peak $\dot{\text{V}}{\text{O}}_{2}$ ($\text{v}\dot{\text{V}}{\text{O}}_{2\text{PEAK}}$) and a final speed of 100% $\text{v}\dot{\text{V}}{\text{O}}_{2\text{PEAK}}$ as suggested by Da Silva et al. [17]. To determine $\text{v}\dot{\text{V}}{\text{O}}_{2\text{PEAK}}$, $\dot{\text{V}}{\text{O}}_{2\text{PEAK}}$ was estimated by a non-exercise model [18] and was imputed in the reverse ACSM metabolic equation for treadmill running [19] to calculate the maximum speed. The warm up was performed at a constant speed of 50% $\text{v}\dot{\text{V}}{\text{O}}_{2\text{PEAK}}$ for three minutes. The treadmill ATL (Inbrasport, Porto Alegre, Brazil) slope was set at 1% during the test and warm-up [20]. Participants were verbally encouraged to perform their maximum effort. After the CPET, participants underwent a passive supine recovery during fifteen minutes. To confirm if the tests were maximum, the participants were required to meet at least three of the following criteria [21]: 1) To achieve volitional fatigue with the maximum RPE score—10 on the Borg CR-10 scale [22]; 2) To achieve an HR ≥ 90% of maximum predicted HR (220-age) or the presence of an HR plateau (ΔHR in two consecutive work rates < 4 beats.min^-1^); 3) To present a plateau of $\Delta \dot{\text{V}}{\text{O}}_{2}$ ($\Delta \dot{\text{V}}{\text{O}}_{2}$ in two consecutive work rates ≤ 2.1 mL.kg^-1^.min^-1^); 4) To achieve a ratio of gas exchange ≥ 1.1.

### Combat simulation procedures

Combat simulation was performed within a 6m x 6m rubber mat area during three rounds of two minutes, with intervals of one minute resting in a seated position, according to official World Taekwondo Federation rules [12]. Participants competed against an opponent within their corresponding weight division and experience. Before the match, participants performed non-contact sparring for three minutes to warm up. To control the warm-up intensity of 40–60% of $\dot{\text{V}}{\text{O}}_{2\text{PEAK}}$ (previously determined in CPET), $\dot{\text{V}}{\text{O}}_{2}$ was continuously measured and the non-contact sparring was administrated by an evaluator, positioned as a centre referee. An official electronic scoring system was used (KP&P, Seoul, South Korea), served by electronic trunk protectors with impact sensors to detect successful midsection techniques. A corner judge was responsible for noting successful head techniques, and a centre referee was responsible for managing the match and to apply penalties [12]. A live score board was publicized during the matches. After the simulation, participants underwent a supine recovery for fifteen minutes.

### Physiological measurements during the simulated combat

**Fig 1:**
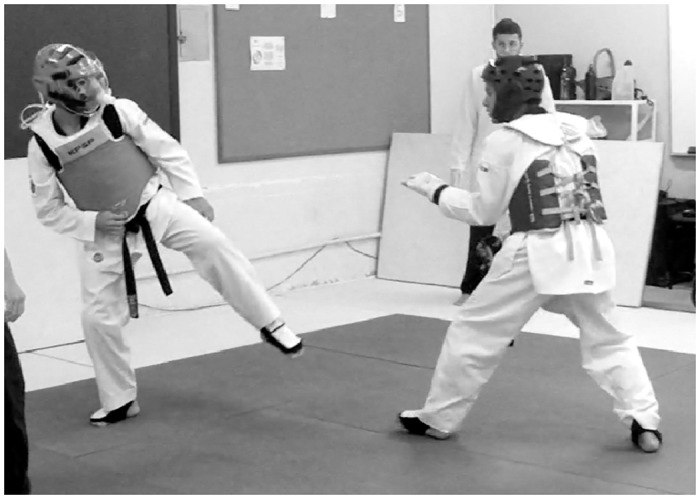
Combat simulation setting.

**Fig 2:**
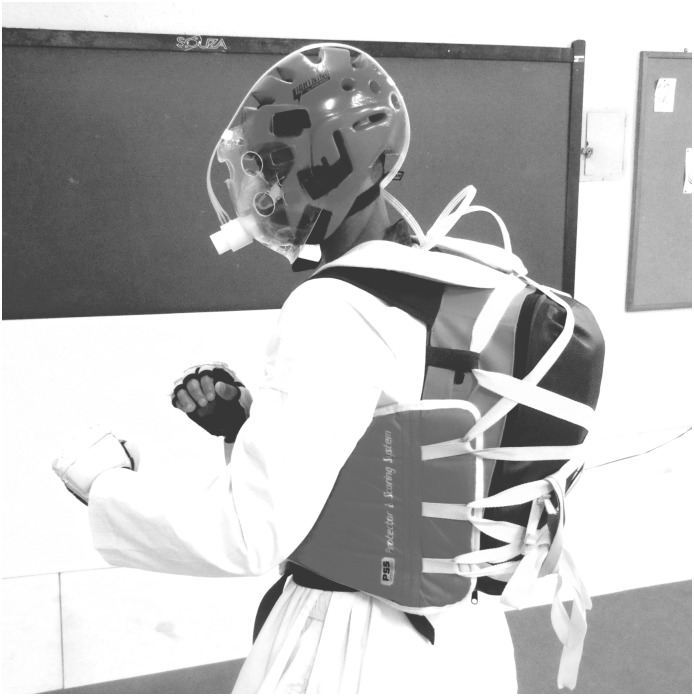
Gas analyser placement during the combat.

$\dot{\text{V}}{\text{O}}_{2}$ was assessed with the VO2000 gas analyser (Medgraphics, Saint Louis, USA), recording at twenty second intervals. Before all $\dot{\text{V}}{\text{O}}_{2}$ measurements, an automatic calibration was performed according to each pneumotachograph. Regarding the gas analyser placement during the combat, a special protector was developed to place the portable analyser safely on the back of the assessed participant, and a plastic pneumotachograph protection was placed in the helmet to protect it from head strikes (Figs 1 and 2). A 7400 Series Vmask silicone oro-nasal mask (Hans Rudolph, Kansas City, USA) was used to collect expired gases in CPET and Competition. Additionally, the acrylic face protection, with multiple air entrances, was attached in the helmet anterior location, guarding the face area, the silicone mask and the pneumotachograph during the combat simulation.

The protector was a lightweight anti-shocking backpack, constructed from polyurethane foam and EVA rubber. Heart rate was assessed with the cardiotachometer RS800cx (Polar Electro, Kempele, Finland), set to record beat-by-beat. Blood lactate was assessed with a validated portable analyser Lactate Plus (Novabiomedical, Waltham, USA) [23]. Blood samples were taken from the participants’ fingertip and were collected immediately after the rest (baseline) and the warm-up, immediately at the end of each round and interval; at the second, fourth, tenth and fifteenth minute of recovery. All equipment was previously calibrated according to manufacturer’s recommendations.

Peak $\dot{\text{V}}{\text{O}}_{2}$ and HR were determined as the highest values verified during the combat situation, and mean $\dot{\text{V}}{\text{O}}_{2}$ and HR were calculated with the average of the round values. Percent values of peak and mean variables were calculated with the peak values _PEAK_ verified in the CPET. Blood lactate response was presented as: [La^-^]PEAK—the highest value detected during the interval after each round; [La^-^]AFTER—the immediate value at the end of the round; and Δ[La^-^]–the difference between the [La^-^] concentration at the immediate end and the beginning of each round. Variables were sampled each round, and the “Total” column represents the sampling of the whole combat, with the subtraction of intervals. To provide a broader understanding of the cardiovascular strain during the combat, the time spent in HR zones was analysed [3,6,24,25]. HR was sampled in mean five seconds values along the three rounds. Then, HR intensity was classified into five zones: A (95%-100%HRmax), B (90–94% HRmax), C (85–89%HRmax), D (84–80% HRmax), E (<80%HRmax).

### External validity

Time-motion analysis was performed to test combat simulation external validity. This technique is widely employed to assess performance of combat bouts, determining effort and recovery time during the matches [1,4,13,26]. Ten athletes provided a video of their last national competition performance containing a full match. Combat simulations and official competition matches were analysed with the Kinovea 0.8.15 software (Joan Charmant & Contributors, Bordeaux, France) with a precision of 0.04 s. Since several Brazilian national competition matches were performed in two rounds only, an option provided by WTF rules [12], combat simulation’s third round was excluded from the analysis. Combat simulations were recorded with a digital video camera Coolpix S8100 (Nikon, Tokyo, Japan).

Performance was assessed by the calculation of average duration of exchange periods (ET), in other words, delimited by the beginning of a kick or punch until both athletes ceased the attack sequences and assumed an active recovery position. The average duration of active recovery position moments, also called preparation time (PT), was also calculated and it was delimited by the end of a previous exchange period until the beginning of the next exchange period, which is generally constituted by bouncing, faints and displacement movements. Exchange/Preparation Ratio and Number of Exchanges were also calculated. Exchange/Preparation Ratio (average ET divided by average PT) is the calculation of the active-passive proportion of the match, and number of exchanges is the frequency of exchange sequences at each round. A single high-experienced and familiarized evaluator performed all the analysis. Half of the competition matches and combat simulations were reanalysed by the same evaluator, and showed a high intraclass correlation (absolute agreement/single measures) for all variables: Preparation Time—ICC: 0,96 (p <0,001); Attack time—ICC 0,97 (p <0,001); ET:PT ratio—ICC: 0,97 (p <0,001); Number of exchange—ICC: 0,95 (p <0,001), agreeing to the time-motion reliability reported in literature [1,4,13].

### Statistical analysis

Statistical analysis was performed using SPSS Statistics for Windows, version 21.0 (IBM, Armonk, USA). Effect size (*ES*) was calculated with G-Power 3.1.9.2 software (Heinrich-Heine-Universität Düsseldorf, Düsseldorf, Germany).

First, Shapiro-Wilk normality test was performed. When normality was confirmed, data were presented as mean, standard deviation, and 95% confidence interval for mean. Between-round comparisons, $\dot{\text{V}}{\text{O}}_{2}$ and HR profiles were performed through repeated-measures ANOVA, with Bonferroni *post hoc* analysis for pairwise comparisons. To test the data sphericity, Mauchly’s test was performed and confirmed it for all parametric variables. When normality was rejected, data were presented as median, interquartile range and 95% confidence interval for median. Between-round comparisons were performed through the Friedman repeated-measures rank test with Dunn *post hoc* analysis for pairwise comparisons. Normality was confirmed for the majority of the dependent variables, except for peak $\dot{\text{V}}{\text{O}}_{2}$ (mL.kg^-1^.min^-1^), mean $\dot{\text{V}}{\text{O}}_{2}$ (mL.kg^-1^.min^-1^), peak HR (%HR_PEAK_), mean HR (%HR_PEAK_) and [La^-^]_PEAK_ (mmol.L^-1^). For a further analysis of $\dot{\text{V}}{\text{O}}_{2}$ and HR, repeated measures ANOVA was performed to verify time-round and time-interval interactions. The proportion of time spent in specific HR intensity zones was also examined non-parametrically using Friedman repeated-measures rank test, with Dunn *post hoc*. Time-motion analysis was examined with paired T-test for total bout analysis, and two-way repeated-measures ANOVA for round (rounds 1 and 2) and condition (competition and simulation) interaction. The alpha level of significance testing was set at *p* < 0.05 for all tests.

The effect size was calculated to assess the difference’s magnitude. Effect size was calculated by Cohen’s *d* (T-test) and *f* (ANOVA), and values were interpreted in accordance to Hopkins et al. [27] as trivial (0–0.2), small (0.2–0.6), moderate (0.6–1.2), large (1.2–2.0) and very large (2.0–4.0). Friedman Test’s effect size was calculated through Kendall’s *W* (*W*), which ranges from 0 (no agreement) to 1 (complete agreement) [28].

## Results

Combat simulation rounds elicited similar peak $\dot{\text{V}}{\text{O}}_{2}$ (*F*_2,22_ = 0.5; *p* = 0.78; *W* = 0.02) and mean $\dot{\text{V}}{\text{O}}_{2}$ (*F*_2,22_ = 3.11; *p* = 0.21; *W* = 0.13) (Table 1). However, heart rate displayed marked variation with peak HR increasing (*F*_2,22_ = 21.8; *p*< 0.001; *ES* = 1.41 [large]) from round 1 to round 2 (*p* = 0.016), and from round 2 to round 3 (*p* = 0.003) (Table 1). Regarding the whole round, mean HR also differed (*F*_2,22_ = 10.8; *p* = 0.001; *ES* = 0.99 [moderate]), with an increase from round 1 to 2 (*p*<0.001) (Table 1). Peak [La^-^] increased significantly (*F*_2,22_ = 18.8; *p*<0.001; *W* = 0.78) between rounds 1 and 3 (p<0.001) (Table 1).

**Table 1:** Cardiorespiratory and blood lactate responses to the simulated taekwondo combat (n = 12).

	Round 1	Round 2	Round 3	Total	*p*-value
**Peak $\dot{\text{V}}{\text{O}}_{2}$*** **(mL.kg**^**-1**^**.min**^**-1**^**)**	42.8±3.8	42.3±5.5	42.3±5.6	44.8±5.0	0.78
	(40.8–45.5)	(35.5–46.7)	(38.6–42.3)	(42.1–46.7)	(0.02^W^)
**Peak $\dot{\text{V}}{\text{O}}_{2}$ ($\%\dot{\text{V}}{\text{O}}_{2\text{PEAK}}$)**	85.5±6.4	84.4±8.4	84.1±7.9	89.1±6.2	0.83
	(81.4–89.5)	(78.9–89.6)	(79.0–89.1)	(85.0–93.0)	(0.13^ES^)
**Mean $\dot{\text{V}}{\text{O}}_{2}$*** **(mL.kg**^**-1**^**.min**^**-1**^**)**	38.1±4.7	36.5±5.1	35.3±3.4	35.9±3.9	0.71
	(35.0–39.4)	(33.3–39.7)	(34.0–38.3)	(34.1–39.1)	(0.13^W^)
**Mean $\dot{\text{V}}{\text{O}}_{2}$ ($\%\dot{\text{V}}{\text{O}}_{2\text{PEAK}}$)**	74.4±6.9	73.0±8.1	72.6±8.0	73.1±6.0	0.70
	(69.9–78.8)	(67.9–78.2)	(66.8–77.4)	(69.3–76.8)	(0.18^ES^)
**Peak HR (beats.min**^**-1**^**)**	181±10^b^^c^	187±14^a^^c^	189±13^a^^b^	190±13	<0.01
	(175–187)	(178–196)	(181–198)	(181–198)	(1.4^ES^)
Peak HR* **(%HR**_**PEAK**_**)**	94.5±6.0^b^^c^	96.0±3.0^a^	97.0±3.0^a^	98.0±3.2	<0.01
	(90.0–96.0)	(95.0–98.0)	(97.0–100.0)	(97.0–100.0)	(0.85^W^)
**Mean HR (beats.min**^**-1**^**)**	173±1^b^^c^	179±11^a^	179±19^a^	177±10	<0.01
	(166–180)	(172–186)	(171–183)	(171–183)	(0.99^ES^)
Mean HR* **(%HR**_**PEAK**_**)**	89.0±6.0^b^^c^	91.7±5.0^a^	93.5±5.0^a^	93.0±5.0	<0.01
	(86.0–92.0)	(90.0–95.0)	(91.0–96.0)	(89.0–94.0)	(0.60^W^)
Peak [La-]* **(mmol.L**^**-1**^**)**	8.4±3.5^c^	10.6±3.4	12.3±2.9^a^	12.6±2.8	<0.01
	(5.5–9.2)	(8.3–12.1)	(10.5–13.4)	(10.5–13.4)	(0.78^W^)
**[La-]**_**AFTER**_ **(mmol.L**^**-1**^**)**	6.7±1.7^b^^c^	8.8±2.5^a^^c^	10.8±2.8^a^^b^	-	<0.01
	(5.5–7.9)	(7.3–10.4)	(9.0–12.5)	-	(1.67^ES^)
**Δ[La-] (mmol.L**^**-1**^**)**	2.7±1.6^c^	1.3±1.2	0.8±1.5^a^	-	<0.01
	(1.7–3.6)	(0.5–2.1)	(-0.1–1.7)	-	(0.73^ES^)

Variables with the absence of asterisk (*) denotes parametric data, presented as mean ± standard deviation and 95% confidence interval (inferior limit—superior limit).

* Denotes non-parametric data, presented as median, interquartile range and 95% confidence interval (inferior limit—superior limit).

Repeated measures ANOVA and Bonferroni Post hoc test outcomes (comparison between rounds):

^a^ Denotes significant different from round 1;

^b^ Denotes significant different from round 2;

^c^ Denotes significant different from round 3. Effect sizes are presented within parentheses, in p-value column:

^*W*^ Denotes Kendall’s *W*;

^*ES*^ Denotes Cohen’s *d* Effect size.

Significant level of *p* < 0.05 adopted for all variables. During cardiopulmonary test, $\dot{\text{V}}{\text{O}}_{2\text{PEAK}}$ and HR_PEAK_ were assessed to express the simulation cardiorespiratory responses in percentage of participants’ peak (e.g. %HR _PEAK_ and $\%\dot{\text{V}}{\text{O}}_{2\text{PEAK}}$). Total column represents the sampling of the whole combat, with the subtraction of intervals.

**Fig 3:**
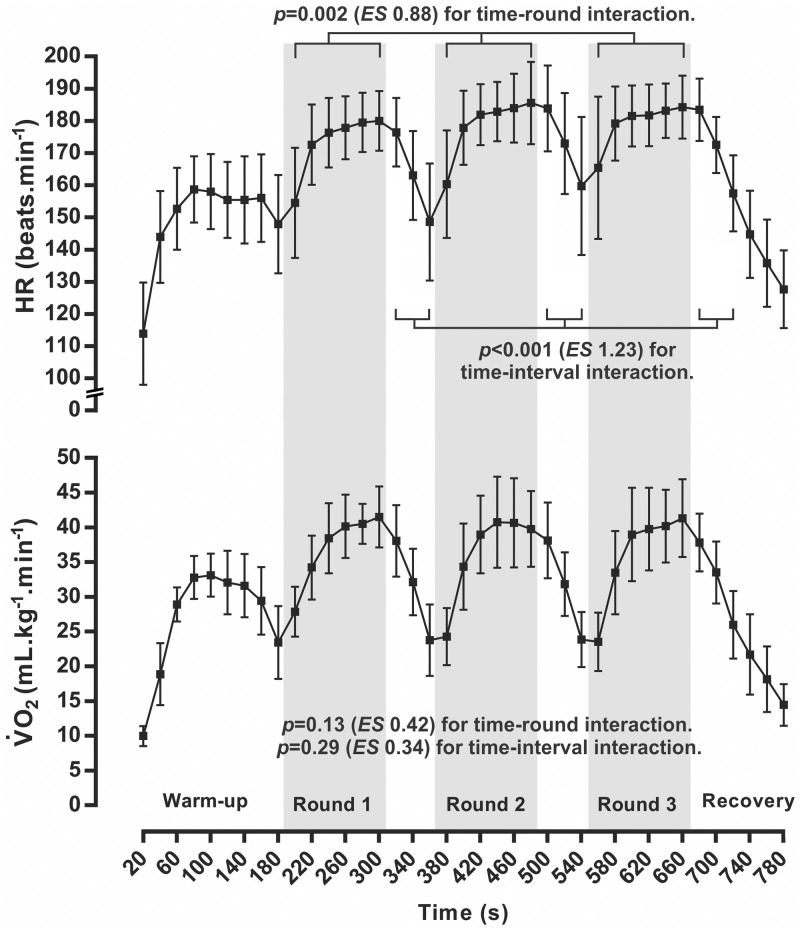
Heart rate (HR) and oxygen uptake ($\dot{\text{V}}{\text{O}}_{2}$) profiles during the combat simulation. Mean values (± standard deviation) of $\dot{\text{V}}{\text{O}}_{2}$ and HR, sampled 20 seconds (HR averaged every 20s) across the simulation. *p–*Significance level for interactions, calculated with repeated measures ANOVA (time x round, or time x interval), and *ES* (effect size) was calculated with Cohen’s *f*.

Detailed HR and $\dot{\text{V}}{\text{O}}_{2}$ profiles during the combat simulation are presented in Fig 3.

**Fig 4:**
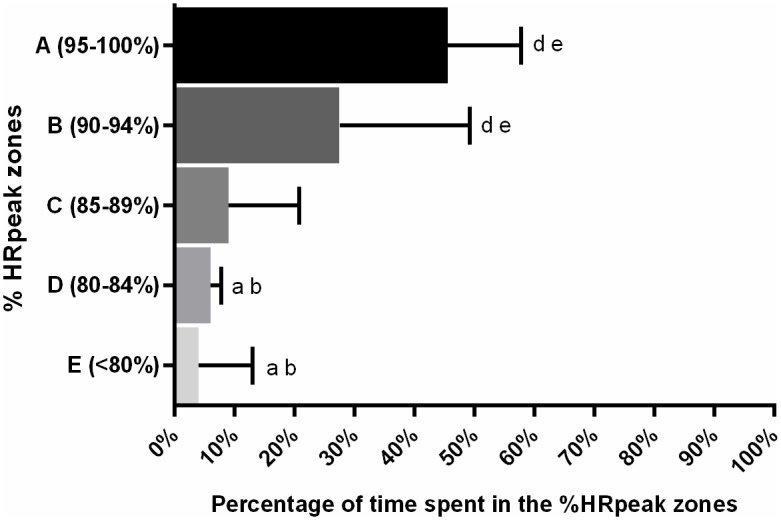
Percentage of time spent in specific heart rate intensity zones during the combat simulation. Significant differences confirmed in Friedman test, with Dunn post-hoc (p<0.05). ^a^ Denotes significant different from the 95–100% zone. ^b^ Denotes significant different from the 90–94% zone. ^d^ Denotes significant different from the 80–84% zone. ^e^ Denotes significant different from the <80% zone.

Further scrutiny of the HR responses during combat revealed that a significantly greater proportion of time was spent in the highest HR intensity zones (*F*_4,44_ = 17.3; *p* = 0.002; *W* = 0.36), with significant higher values in zones A (>95% HR_PEAK_) and B (90–94% HR_PEAK_) compared to D (80–84% HR_PEAK_) and E (<80% HR_PEAK_) (*p* < 0.05, for all respective differences) (Fig 4).

Time-motion analysis for total match (n = 10) indicated a shorter preparation time (*p* = 0.01; *ES* = 1.11 [moderate]) and lower ET:PT ratio (*p* = 0.02; *ES* = 0.86 [moderate]) along the rounds in combat simulation (Table 2).

**Table 2:** Time-motion analysis of competition matches and combat simulations (n = 10).

	Round 1		Round 2		Total	
	Comp	Simul	Comp	Simul	Comp	Simul
**Exchange time (s)**	2.1±0.8	2.0±0.5	2.0±0.4	2.2±0.6	2.0±0.5	2.1±0.5
	(1.7–2.6)	(1.6–2.5)	(1.6–2.3)	(1.9–2.6)	(1.7–2.4)	(1.7–2.5)
**Preparation time (s)**	5.2±1.7^†^	3.2±1.3^†^	4.2±1.2^†^	3.2±1.3^†^	4.6±1.2*	3.2±1.3*
	(4.1–6.2)	(2.2–4.2)	(3.4–5.0)	(2.3–4.2)	(3.7–5.5)	(2.3–4.1)
**ET:PT ratio (1:x)**	2.8±1.4^†^	1.8±1.2^†^	2.2±0.8^†^	1.6±0.7^†^	2.4±0.9*	1.7±0.9*
	(1.9–3.7)	(0.9–2.7)	(1.7–2.7)	(1.0–2.1)	(1.8–3.1)	(1.0–2.3)
**Number of exchanges**	16±4	20±4	20±4	19±6	36±7	39±9
	(14–19)	(17–22)	(16–23)	(16–23)	(31–1)	(33–46)

Comp—Competition Match. Simul—Combat Simulation. ET:PT ratio—Exchange Time/Preparation Time ratio. Total—Average of the two rounds. Variables presented as mean ± standard deviation, and 95% confidence interval (inferior limit—superior limit).

Two-way repeated-measures ANOVA (condition x round) outcome:

^**†**^ Denotes significant difference in condition (Competition match and Combat simulation) and round (Round 1 and Round 2).

Paired T-test outcome:

*Denotes significant difference for condition factor (Competition match and Combat simulation).

Significant level of *p* < 0.05 adopted for all variables.

## Discussion

This is the first study to assess the cardiorespiratory responses to a full-contact taekwondo combat whilst also capturing the full-contact competitive elements of the bout, including the use of official electronic scoring system sensors, and a free full-contact interaction with a competitive opponent. The anti-shock protection, designed specifically for the portable gas analyser, was successful in ensuring the integrity of the cardiorespiratory measurements whilst also permitting suitable competitiveness during the bouts. Time-motion analysis demonstrated broadly similar activity profiles between the simulation and competition, suggesting acceptable external validity of mechanical actions performed in the simulated combat. The competitive full-contact combat simulation elicited moderate-to-high cardiorespiratory responses and high blood lactate concentrations, suggesting marked participation from both aerobic and anaerobic metabolic pathways in this setting.

Combat simulation demanded a high cardiovascular strain, with a significant increase in peak heart rate across the rounds. Regarding the entire round, mean heart rate indicated that rounds 2 and 3 elicited a higher demand than round 1. Such responses have also been documented in international competition [3,24], suggesting that athletes must be able to sustain the increasing demands of the combat and still produce explosive strikes to score, and fast displacements and defensive reactions to achieve the victory. Curiously, such differences in the cardiorespiratory responses across the rounds were not accompanied by a difference in the time-motion variables. Nevertheless, the time spent in the highest HR zones (>90% HR_PEAK_) agreed to international championships [3,24], with a predominance of 75% of total time. Such high cardiovascular demands corroborate the importance of aerobic power to succeed in taekwondo performance [4].

In contrast to the elevated HR response, oxygen consumption did not increase across the match. Despite the well documented relationship between HR and $\dot{\text{V}}{\text{O}}_{2}$ during exercise [29], combat simulation presented a marked dissociation between the responses profiles, which needs to be elucidated. All rounds presented similar contributions from the oxidative system, which might reflect the substrate restoration, e.g. ATP turnover, and cyclic preparation movements such as bouncing and displacements [4]. Other simulations have attempted to measure oxygen consumption during simulated combat [4,13]. Campos et al. [4] presented a different profile, with a significant lower mean $\dot{\text{V}}{\text{O}}_{2}$ in first round, compared to the next two rounds. Lopes-Silva et al. [13] also found a different behaviour, with an increase from first to second round, followed by a decrease from second to third. Nevertheless, the absence of percentage values of $\dot{\text{V}}{\text{O}}_{2}$ in the literature precludes further comparisons. Concerning ecological validity, simulations comparison must consider factors such as the a presence or absence of protective gear to the gas analyser of an official electronic scoring system, the level aerobic fitness and tactical style of the samples, and the use of different measurement systems.

Regarding the anaerobic responses, combat simulation elicited a moderate-to-high blood lactate response, achieving a peak of 12.3 mmol.L^-1^. A crescent response profile was observed across the rounds, suggesting marked activation of glycolysis during the match. The Δ[La-] in first round denotes that anaerobic glycolysis was already elicited at the beginning of the bout. Such blood lactate profile agreed with international competition [3,24]. Bridge et al. [3] suggested this lactate response can be associated with an augmented adrenaline-mediated activation of glycolysis and glycogenolysis by increased glycogen phosphorylase activity elicited during the competition bout. Therefore, competitiveness seems to be an important aspect to activate this metabolic pathway to support explosive strikes, faints, blocks and displacements required during competition.

Time-motion analysis has been presented as a solution to assess the demands of official matches. Taekwondo athletes performed a simulated match with similar exchange time and number of exchanges to official competition. The simulation equipment and combative setting did not reduce competitiveness with a longer preparation time. Also during the combat simulation, Lopes-Silva et al. [13] presented a recent report of exchange-preparation ratio close to 1:2, agreeing with the present simulation. Earlier studies presented an exchange-preparation ratio ranging between 1:4 and 1:7 [1–3], which might be explained by the recent WTF taekwondo rule updates, such as electronic impact scoring system, 10s preparation limit, and more rewards to spinning and head techniques [12]. Despite a shorter preparation time, the current simulation provided a reasonable approximation of activity performed in actual competition and cardiorespiratory and blood lactate responses provided a reasonably accurate depiction of the metabolic responses during the event.

The findings of the present study detail the importance of both aerobic and anaerobic metabolic pathways during competitive taekwondo matches and training sessions. The conditioning program also needs to consider the anaerobic participation to succeed across the rounds, which is important to keep a scoring advantage or to reverse a disadvantage. Furthermore, combat sessions could be controlled by cardiorespiratory responses, and manipulated by round and interval durations; attacking and preparation durations; or kicking frequency. However, the efficacy of combat training as a stimulus for the enhancement of both aerobic and anaerobic capacities still needs to be elucidated. Mixing conditioning and tactical training in a single session seems to be an efficient strategy to improve taekwondo athletes performance, since the tactical component can be developed together with the cardiorespiratory fitness; it may optimize the training volume and save energy expenditure for other sessions or to a proper recovery. Besides taekwondo, several combat sports are facing the same challenge in assessing oxygen consumption during a simulation. Crisafuli et al. [30] proposed a dynamic sequence of Muay Thai strikes on a pad, conducted by an experienced coach. Doria et al. [31] and Beneke et al. [32] performed a simulated karate match (kumite) with a similar method to Campos et al. [4], i.e., a sparring between an assessed athlete (carrying the portable gas analyzer) and an opponent. Therefore, our simulation method could also be employed in theses modalities to enhance the external validity of cardiorespiratory assessments.

The present combat simulation setting was successful in permitting $\dot{\text{V}}{\text{O}}_{2}$ measures during a full-contact taekwondo contest using the official scoring system. The gas analyser protection provided a safe measurement of gas exchanges during combat and the new simulation setting elicited moderate to high cardiorespiratory demands on the competitors. Combative strategies, such as the referee participation and electronic scoring system also assisted to provide a reasonable approximation of activity performed in actual competition. Practically, the findings of the present study affirm the importance of both aerobic and anaerobic metabolic pathways in competitive taekwondo, establishing a contemporary and more externally valid ergonomic framework for taekwondo training. Additionally, several efforts have been made to improve the specificity of aerobic profile testing [33] and competition-specific conditioning [34]. These advances may collectively serve to enhance scientific preparatory strategies within the sport. The current framework might also be considered by other researchers investigating the metabolic demands of taekwondo and/or other combat sports to improve the external validity of their measurements and hence optimize preparations for competition.
