# Correction: Physiological responses and external validity of a new setting for taekwondo combat simulation

**DOI:** 10.1371/journal.pone.0181298

**Published:** 2017-07-07

**Authors:** Matheus Hausen, Pedro Paulo Soares, Marcus Paulo Araújo, Flávia Porto, Emerson Franchini, Craig Alan Bridge, Jonas Gurgel

There is an error in the ninth sentence of the Abstract. The correct sentence is: A peak $\dot{V}O_{2}$ of 44.8 ± 5.0 ml.kg^-1^.min^-1^ (89 ± 5% $\dot{V}O_{2\mathrm{PEAK}}$), a peak heart rate of 190 ± 13 beats.min^-1^ (98 ± 3% HR_PEAK_) and peak [La^-^] of 12.3 ± 2.9 mmol.L^–1^ was elicited by the bouts.

There are errors in the last sentence of the third paragraph of the “Physiological measurements during the simulated combat” section of the Methods [1]. The correct sentence is: Then, HR intensity was classified into five zones: A (95%-100%HR_PEAK_), B (90–94% HR_PEAK_), C (85–89%HR_PEAK_), D (84–80% HR_PEAK_), E (<80%HR_PEAK_).

In Table 2, the value in the Total Comp column and Number of exchanges line should be 31–41. There is an error in Table 2 caption for Two-way repeated-measures ANOVA outcome. The correct sentence is: ^**†**^ Denotes significant difference for condition factor (Competition match and Combat simulation). Please see the corrected Table 2 here.

**Table 2:** Time-motion analysis of competition matches and combat simulations (n = 10).

	Round 1		Round 2		Total	
	Comp	Simul	Comp	Simul	Comp	Simul
**Exchange time (s)**	2.1±0.8	2.0±0.5	2.0±0.4	2.2±0.6	2.0±0.5	2.1±0.5
	(1.7–2.6)	(1.6–2.5)	(1.6–2.3)	(1.9–2.6)	(1.7–2.4)	(1.7–2.5)
**Preparation time (s)**	5.2±1.7^†^	3.2±1.3^†^	4.2±1.2^†^	3.2±1.3^†^	4.6±1.2*	3.2±1.3*
	(4.1–6.2)	(2.2–4.2)	(3.4–5.0)	(2.3–4.2)	(3.7–5.5)	(2.3–4.1)
**ET:PT ratio (1:x)**	2.8±1.4^†^	1.8±1.2^†^	2.2±0.8^†^	1.6±0.7^†^	2.4±0.9*	1.7±0.9*
	(1.9–3.7)	(0.9–2.7)	(1.7–2.7)	(1.0–2.1)	(1.8–3.1)	(1.0–2.3)
**Number of exchanges**	16±4	20±4	20±4	19±6	36±7	39±9
	(14–19)	(17–22)	(16–23)	(16–23)	(31–41)	(33–46)

Comp–Competition Match. Simul–Combat Simulation. ET:PT ratio—Exchange Time/Preparation Time ratio. Total–Average of the two rounds. Variables presented as mean ± standard deviation, and 95% confidence interval (inferior limit–superior limit).

Two-way repeated-measures ANOVA (condition x round) outcome:

^**†**^ Denotes significant difference for condition factor (Competition match and Combat simulation).

Paired T-test outcome:

*Denotes significant difference in condition (Competition match and Combat simulation).

Significant level of *p* < 0.05 adopted for all variables.
